# Regenerable Poly(dopamine)-Mediated Gold Nanostructure-Decorated Core–Shell Nanostructures of Magnetite/Polydopamine for Catalytic Dye Removal

**DOI:** 10.1021/acsomega.4c06968

**Published:** 2024-12-18

**Authors:** Nuray Serginay, Mehmet Semih Bingol, Erkan Karatas, Mehmet Yilmaz

**Affiliations:** † Department of Nanoscience and Nanoengineering, Atatürk University, Erzurum 25030, Turkiye; ‡ East Anatolia High Technology Application and Research Center, Atatürk University, Erzurum 25030, Turkiye; § Department of Molecular Biology and Genetics, Erzurum Technical University, Erzurum 25100, Turkiye; ∥ Department of Chemical Engineering, Atatürk University, Erzurum 25030, Turkiye

## Abstract

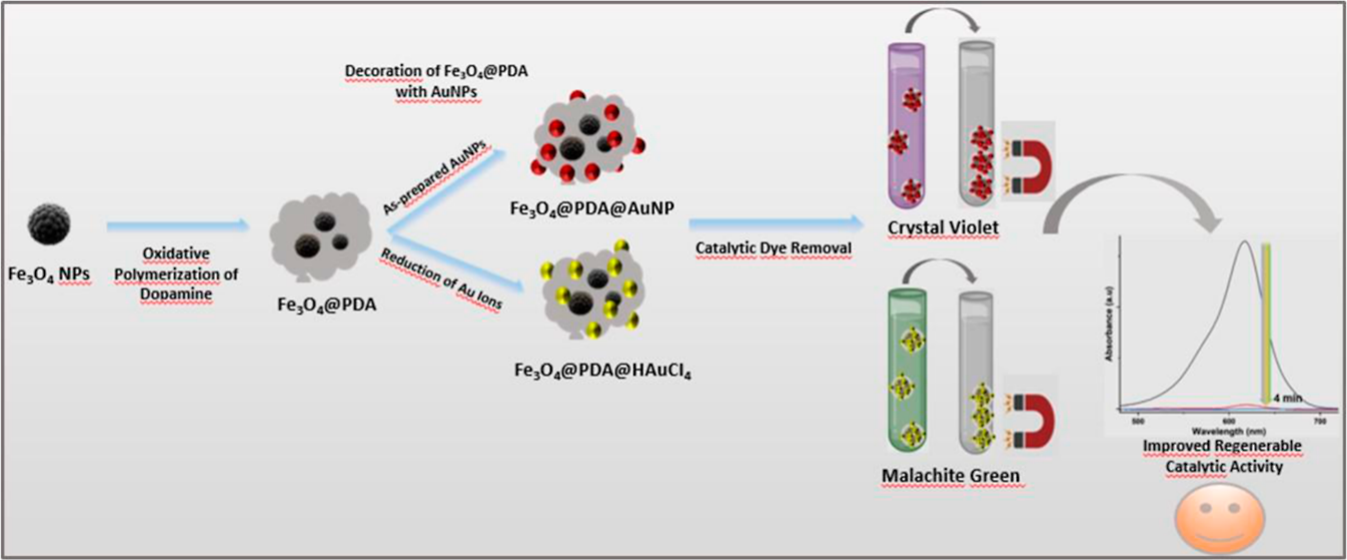

In this paper, we present a facile yet effective method for the fabrication of core–shell nanoparticles (NPs) of magnetite (Fe_3_O_4_) and polydopamine (Fe_3_O_4_@PDA) and their decoration with a tunable amount of gold NPs (AuNPs). For this, Fe_3_O_4_ NPs were fabricated through the polyol method and AuNPs were deposited onto Fe_3_O_4_@PDA via anchoring of as-prepared citrate-stabilized AuNPs or reduction of Au ions. PDA with its numerous catechol groups enabled the decoration of AuNPs in a well-controlled manner. The resultant Fe_3_O_4_@PDA@Au nanosystem exhibited highly efficient catalytic activity in removing crystal violet (CV) and malachite green (MG) as dye molecules. It was noticed that the quantity of deposited AuNPs was the primary determinant of the resulting catalytic activity of the suggested system. Both techniques resulted in NP systems demonstrating distinct catalytic activity with reaction constant values of 0.83 and 1.54 min^–1^ for removing CV and MG dyes, respectively. The complete dye removals were attained only within 4 min. Furthermore, the core–shell nanosystem was easily regenerated by removing it from the medium via an external magnet and subsequent washing. Even after five cycles, the catalytic system provided satisfying activity in both dyes indicating its high reusability capacity. The combination of AuNPs with distinct characteristics of PDA and magnetic NPs makes this core–shell nanosystem a viable platform for various catalytic and wastewater applications.

## 1 Introduction

Water contamination due to organic pollutants has emerged as a critical environmental problem and has garnered much attention.^1−4^ Addressing the pollution issues originating from organic dyes is particularly crucial as a result of their extensive use in several industries including printing, textile, paper, paints, and plastics.^5,6^ The excessive discharge of wastewater containing organic dyes can hinder the penetration of sunlight into water, leading to a decrease in the photosynthetic activity of plants.^7^ Additionally, the accumulation of organic dyes in the human body through drinking water can create a detrimental effect on human health and lead to many disorders, such as respiratory damage, genetic mutations, immune system suppression, and cancer.^8−10^ Immediate action is necessary to implement effective strategies for the elimination of organic pollutants from natural water sources. Various strategies have been utilized to eliminate these pollutants, including adsorption, photocatalytic degradation, chemical oxidation, membrane filtering, flocculation, and electro-oxidation.^11−15^

Adsorption is a highly efficient method for treating organic dyes, widely employed due to its cost-effectiveness and simplicity.^16,17^ A range of adsorbents, such as activated carbon, carbon nanotubes, graphene hydrogels, and metal oxide, have been developed to effectively eliminate dyes from water through strong adsorption properties.^5,18−20^ In addition to adsorption, chemical reduction is a convenient and efficient approach for removing organic dyes in aqueous solutions. The intricate harmful organic pollutants can be converted into nonhazardous derivatives through chemical structure alteration or further decomposed into nontoxic tiny molecules.^21^ The chemical reduction process can occur only in the presence of appropriate catalysts. Extensive study has been conducted on several catalysts used for the catalytic reduction of diverse organic dye contaminants.^6^ However, the majority of catalysts focus on selectively reducing a single organic dye, leading to limited effectiveness in removing complex organic pollutants, particularly when multiple organic pollutants are simultaneously released into water. Therefore, the development of a comprehensive catalyst can effectively reduce complex organic dye contaminants. Lately, there has been a significant focus on the use of tailored and well-designed heterogeneous catalysts, which has generated a lot of interest among researchers.^22^ Heterogeneous catalysts have demonstrated superiority compared to homogeneous catalysts in terms of their ease of separation from the reaction mixture, mild reaction conditions, avoidance of tedious workup procedures, and most importantly, the ability to regenerate and reuse the catalysts without any preactivations.^23−26^ Additionally, there is no significant loss of catalytic activity or leaching during subsequent cycles.

Gold nanoparticles (AuNPs) as heterogeneous catalysts have garnered significant interest due to their distinct catalytic characteristics which rely on their negative redox potential.^27−32^ With these unique properties, they have been employed in various oxidation and reduction applications.^33^ Typically, AuNPs in the liquid phase have a strong inclination to aggregate as a result of their high surface energy. This aggregation results in a reduction in their catalytic activity.^34^ Various solid matrices, including carbon nanotubes, silica, titania, and other metal oxides, have been employed as supports for AuNPs in order to improve the stability of the catalyst.^35,36^ Nevertheless, the process of isolating these catalysts from the reaction system requires time-consuming separation techniques such as filtration or centrifugation.^27^ Recently, core/shell nanostructures of magnetite (Fe_3_O_4_) and polydopamine (PDA) (Fe_3_O_4_@PDA) have been utilized as a support material for plasmonic metal NPs and used in various catalytic dye removal applications.^3,5,6,22,27^ PDA, inspired by the adhesive protein of marine mussels, can be deposited conformally onto various materials simply under weak alkaline conditions via oxidative polymerization of dopamine (DA).^30,37−44^ With its numerous catechol groups, PDA can reduce various metal ions and attach NPs with high stability without using any reducing agent, seed material, or stabilizer. On the other hand, Fe_3_O_4_ NP as a core material enables the regeneration and reuse of the heterogeneous catalyst.^45,46^ Despite the significant progress in this research field, novel strategies are still highly demanded to obtain metallic NP-decorated Fe_3_O_4_@PDA nanosystems in a well-controlled manner in various catalytic applications.

In this study, for the first time, we have proposed a novel approach to fabricate AuNP-decorated Fe_3_O_4_@PDA nanosystems (Fe_3_O_4_@PDA@Au) as a robust and highly regenerable heterogeneous catalyst for the removal of some dyes. For this, as-prepared Fe_3_O_4_ NPs were added to a suspension of PDA NPs during their synthesis. This procedure created Fe_3_O_4_@PDA nanosystems with a flower-bud-shape morphology. For the decoration of AuNPs, two novel different strategies were employed: the usage of as-prepared citrate-stabilized AuNPs or the reduction of Au ions. The Fe_3_O_4_@PDA@Au nanosystem presents these outstanding features: (i) the nanosystem can be readily separated by utilizing an external magnet because of the magnetic nature of Fe_3_O_4_ NPs. (ii) The PDA shell acts as a support material to attach as-prepared AuNPs or a reducing agent for Au ions. (iii) The PDA layer provided high adsorbance against oppositely charged dye molecules. (iv) The number of deposited AuNPs was easily manipulated by tuning the experimental parameters, which dramatically determined the catalytic activity of the resultant nanosystem in the removal of crystal violet (CV) and malachite green (MG) as dye molecules. The proposed study provides significant novelty in terms of the NP synthesis route, well-controlled Au nanostructure decoration, and remarkably high catalytic activity.

## 2 Experimental Section

### 2.1 Materials

Iron(III) chloride hexahydrate (FeCl_3_·6H_2_O), DA, chloro auric acid (HAuCl_4_), sodium borohydride (NaBH_4_), CV, MG, succinic acid, propylene glycol, and urea were purchased from Sigma-Aldrich and employed as received. All materials were purchased from Sigma-Aldrich. In all experiments, deionized (DI) water was used.

### 2.2 Fabrication of Fe_3_O_4_, Fe_3_O_4_@PDA, and PDA NPs

**Scheme 1:**
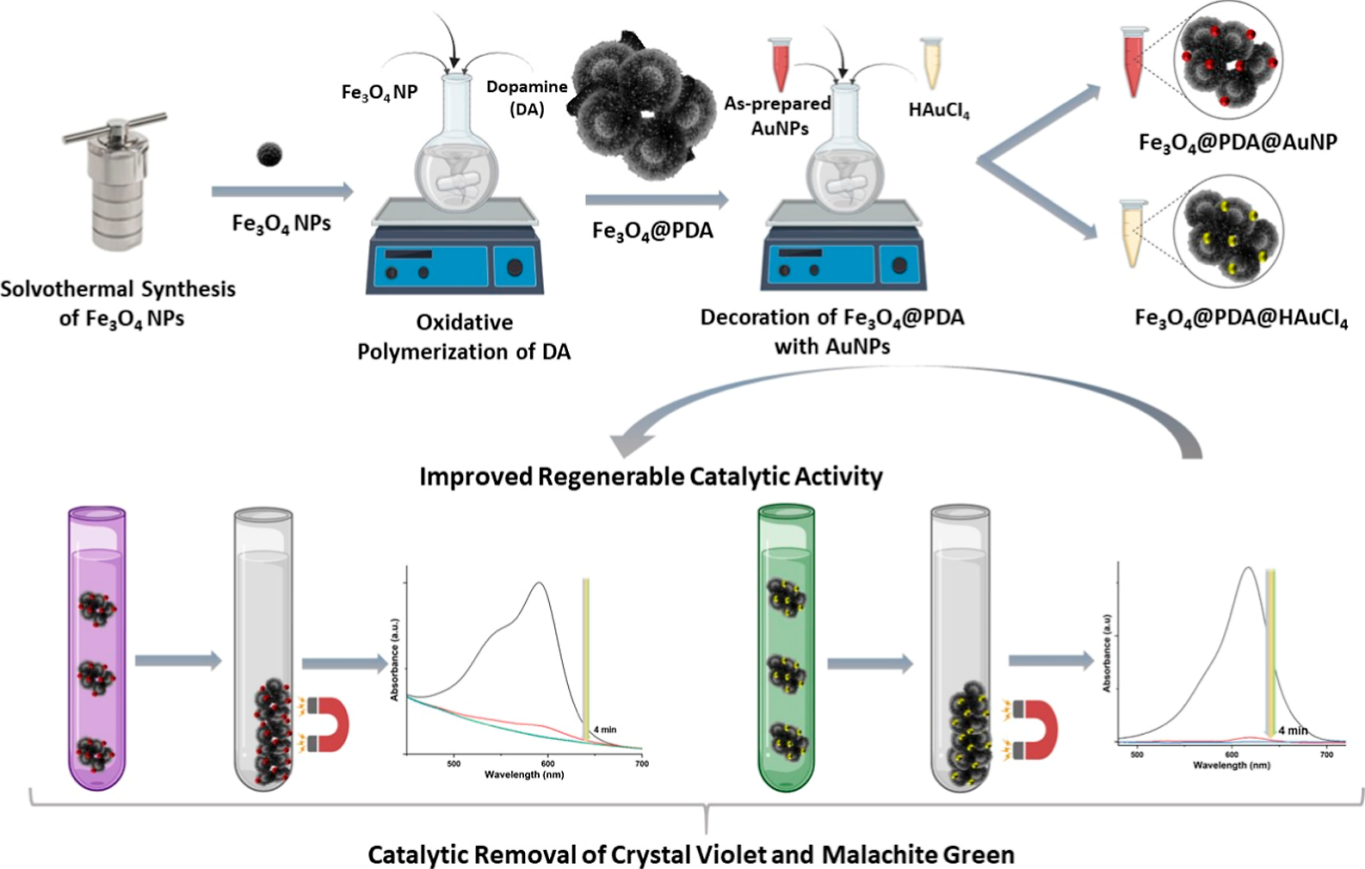
Summary of Experimental Procedures Performed in This Study

We employed the polyol method (see Scheme 1) developed by Cheng et al.^47^ for the synthesis of Fe_3_O_4_ NPs with some modifications. For this, 30 mmol of urea, 3 mmol of FeCl_3_·6H_2_O, and 1 mmol of succinic acid were mixed in 30 mL of propylene glycol and mixed until completely dissolved. After complete solvation, the transparent bright-yellow solution was transferred into a 50 mL TEFLON-stainless steel reactor. The reactor was placed in an oven at 200 °C for 12 h. To terminate the reaction, the oven was turned off and the reactor was kept at ambient conditions to cool to room temperature. The resultant Fe_3_O_4_ (magnetite) NPs were collected by washing consecutively many times with ethanol and DI water by using a strong magnet.

For the fabrication of the core–shell Fe_3_O_4_@PDA nanosystem, we proposed a novel approach by combining the as-prepared Fe_3_O_4_ and PDA NPs (see Scheme 1). For this, first, 40 mg of DA was dissolved in 20 mL of 10 mM Tris-Buffer solution at pH 10 to initiate the growth of PDA NPs. The oxidative polymerization DA was confirmed through the change in the color of the solution from colorless to brownish gray. After 3 h of magnetic stirring at 3000 rpm, 5 mg of Fe_3_O_4_ NP was added to this suspension and stirred for 21 h. Finally, Fe_3_O_4_@PDA NPs were collected by using a magnet and centrifugation (10,000 rpm for 20 min). The resultant Fe_3_O_4_@PDA nanosuspension in dark-brown color was redispersed in DI water and stored under ambient conditions until further use. For the comparison, we also fabricated PDA NPs through the same procedure.

### 2.3 Fabrication of AuNPs, Fe_3_O_4_@PDA@AuNP, and Fe_3_O_4_@PDA@HAuCl_4_ Nanosystems

We employed the citrate-reduction method for the synthesis of AuNPs similar to our earlier reports.^30,38,40,42,48,49^ Briefly, in a round-bottom flask, 50 mL of 1 mM aqueous HAuCl_4_ solution was heated to boiling under vigorous magnetic stirring. Then, an appropriate amount of trisodium citrate solution (0.1 M, 1.65 mL) was added to the boiling mixture to reduce the gold ions. After 5 min, the heater was turned off. The AuNPs with ruby-red suspension were cooled to room temperature and purified via centrifugation at 14,000 rpm for 20 min and redispersed in DI water. For the decoration of AuNPs onto Fe_3_O_4_@PDA NPs (Fe_3_O_4_@PDA@Au), we tested two approaches to determine a nanosystem with higher photocatalytic activity. In the first strategy, a fixed amount (2 mg in 2 mL of DI water) of Fe_3_O_4_@PDA nanosuspension was mixed with different amounts of as-prepared AuNP suspension (80 ppm). For 160, 800, and 1000 μL of AuNP, the resultant Fe_3_O_4_@PDA@Au nanosystems were denoted as Fe_3_O_4_@PDA@AuNP-l, Fe_3_O_4_@PDA@AuNP-m, and Fe_3_O_4_@PDA@AuNP-h, respectively. The AuNP deposition was terminated by centrifugation at 10,000 rpm for 20 min after 3 h of interaction time. In the second strategy, a fixed amount (2 mg in 2 mL of DI water) of Fe_3_O_4_@PDA nanosuspension was mixed with different amounts of chloroauric acid solution at a fixed concentration (1 mM). In a similar vein, the reduction of Au ions was terminated by centrifugation at 10,000 rpm for 20 min after 3 h of interaction time. For 20, 40, and 100 μL of HAuCl_4_, the resultant Fe_3_O_4_@PDA@Au nanosystems were denoted as Fe_3_O_4_@PDA@HAuCl_4_-l, Fe_3_O_4_@PDA@HAuCl_4_-m, and Fe_3_O_4_@PDA@HAuCl_4_-h, respectively. Herein, it must be noted that PDA with its numerous catechol and amine groups could capture and deposit AuNPs as well as reduce Au ions without using any reducing agent and stabilizer on the surface of Fe_3_O_4_@PDA.^30−32,37−42,44,50−53^

### 2.4 Characterization of NPs

The morphology and size of each NP system were evaluated using transmission electron microscopy (TEM) of a Hitachi HighTech HT7700 operated at 100 kV. Also, EDX (energy-dispersive X-ray) spectra were collected to determine the elemental content of the NP systems. We also used a UV–vis spectrophotometer (Shimadzu, UV-1800) to determine the optical properties of the NPs. The crystalline phase of the nanosystems was determined by an X-ray diffractometer (PANalytical Empyrean). FT-IR (Bruker VERTEX 70v) analysis was performed for their functional groups. A Micromeritics 3Flex device was employed to determine the surface area, pore volume, and average pore diameter through Brunauer–Emmett–Teller (BET) analysis.

### 2.5 Catalytic Activity Tests

To evaluate the photocatalytic activity of each NP system, we performed various tests in the photocatalytic degradation of CV and MG. In a typical catalytic activity assay, 1 mL of NP suspension (100 ppm) was added to 3 mL of a mixture of CV and NaBH_4_ to obtain their final concentrations as 1 × 10^–4^ and 0.033 M, respectively. The catalytic conversion of CV was monitored by collecting UV–vis absorption spectra (Shimadzu, UV-1800) at different time intervals. The catalytic conversion resulted in the disappearance of the absorption band of CV centered at around 590 nm. After obtaining the nanosystem with the highest performance, the photocatalytic degradation of CV and MG was investigated for stability and reusability. After each cycle, the NPs were collected via a magnet and washed with DI water for regeneration.

## 3 Results and Discussion

### 3.1 Characterization of NPs

**Figure 1:**
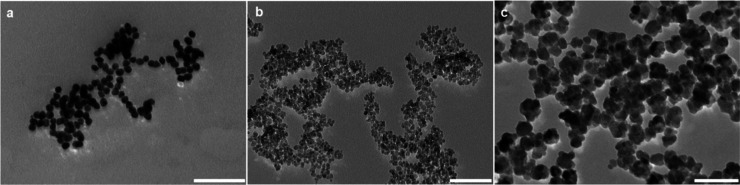
TEM images of AuNPs (a), Fe_3_O_4_ NPs (b), and Fe_3_O_4_@PDA nanosystems (c). Scale bars are 100 nm.

Before characterization of Fe_3_O_4_@PDA@Au nanosystems, we performed a detailed TEM analysis to evaluate the morphology of AuNPs, Fe_3_O_4_, Fe_3_O_4_@PDA, and PDA nanosystems. For each NP system, a representative image is provided in Figures 1 and S1. The citrate-reduction method led to the emergence of AuNPs in spherical morphology (Figure 1a), with particle size in the range 15–26 nm and an average size of 19.8 nm (Figure S3a). The plasmonic nature of AuNP suspension created its unique ruby-red color and resultant absorption maxima at 519 nm (Figure S2).^38,40,42,48^ Fe_3_O_4_ NPs with highly porous structures in different shapes (Figure 1b) were fabricated via the polyol process. Their particle sizes ranged from 8 to 16 nm with an average size of 12.1 nm (Figure S3b). For comparison, we also fabricated bare PDA NPs. The TEM image (Figure S1) and particle size analysis (Figure S3c) for these NPs indicated a flower-bud-shape morphology in the range of 46–80 nm and with an average size of 64.8 nm. The addition of Fe_3_O_4_ NPs into the suspension during the synthesis of PDA NPs resulted in the core/shell systems of Fe_3_O_4_ and PDA (see Figure 1c). The final morphology and size range of Fe_3_O_4_@PDA nanosystems were similar to those of PDA NPs.

**Figure 2:**
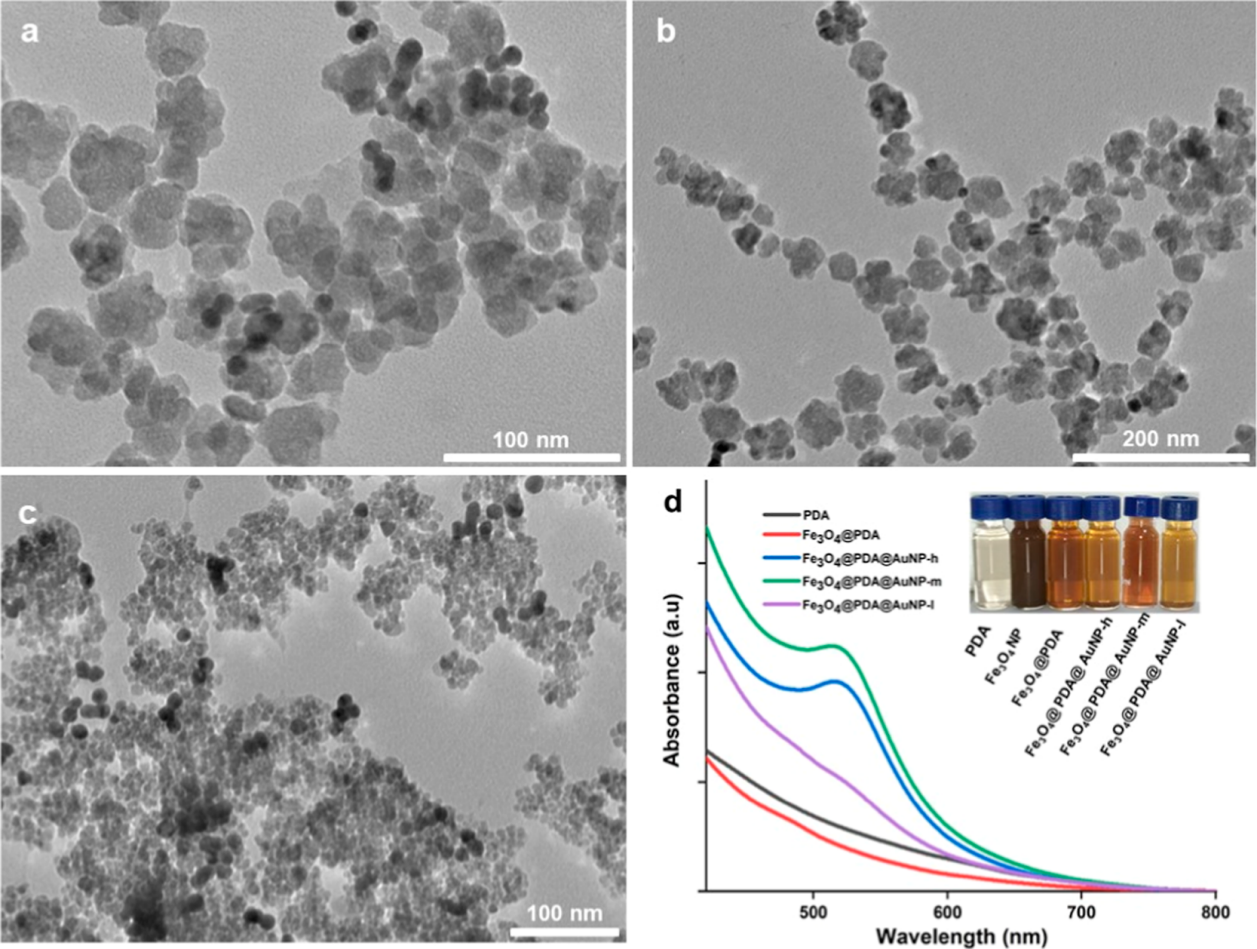
TEM images of Fe_3_O_4_@PDA@AuNP-l (a), Fe_3_O_4_@PDA@AuNP-m (b), and Fe_3_O_4_@PDA@AuNP-h (c) and UV–vis absorption spectra (d) of each nanosystem. The inset shows the optical images of NP suspensions.

The employment of AuNPs with high stability and reusability is essential in photocatalytic dye removal applications. To manipulate the deposition of AuNPs onto the Fe_3_O_4_@PDA nanosystems, we utilized two different strategies. In the first strategy, as-prepared citrate-stabilized AuNPs in different quantities were combined with Fe_3_O_4_@PDA NPs. For each case, TEM images (Figure 2a–c) and absorption spectra (Figure 2d) of the resulting suspension were collected. After the centrifugation as a purification step, the color of the suspension was dramatically tuned (see the inset of Figure 2d) and detected by even the naked eye. For all cases, the deposition of AuNPs onto Fe_3_O_4_@PDA was observed indicating the strong binding affinity of PDA against metallic NPs due to its abundant catechol functional groups.^30−32,38,40−42,50,52−54^ TEM images (Figure 2a–c) indicated that as the amount of supplied AuNPs was increased, the number of deposited NPs increased accordingly. This issue was further confirmed by absorption spectra (Figure 2d). For the case of a low supply of AuNPs (Fe_3_O_4_@PDA@AuNP-l), a weak shoulder was observed at around 520 nm. However, the increase in the amount of plasmonic metal (Fe_3_O_4_@PDA@AuNP-m and Fe_3_O_4_@PDA@AuNP-h) led to the emergence of a distinctive absorption band at around 530 nm. The number of deposited AuNPs was highly correlated with its supply and could be easily manipulated by tuning the experimental parameters.

**Figure 3:**
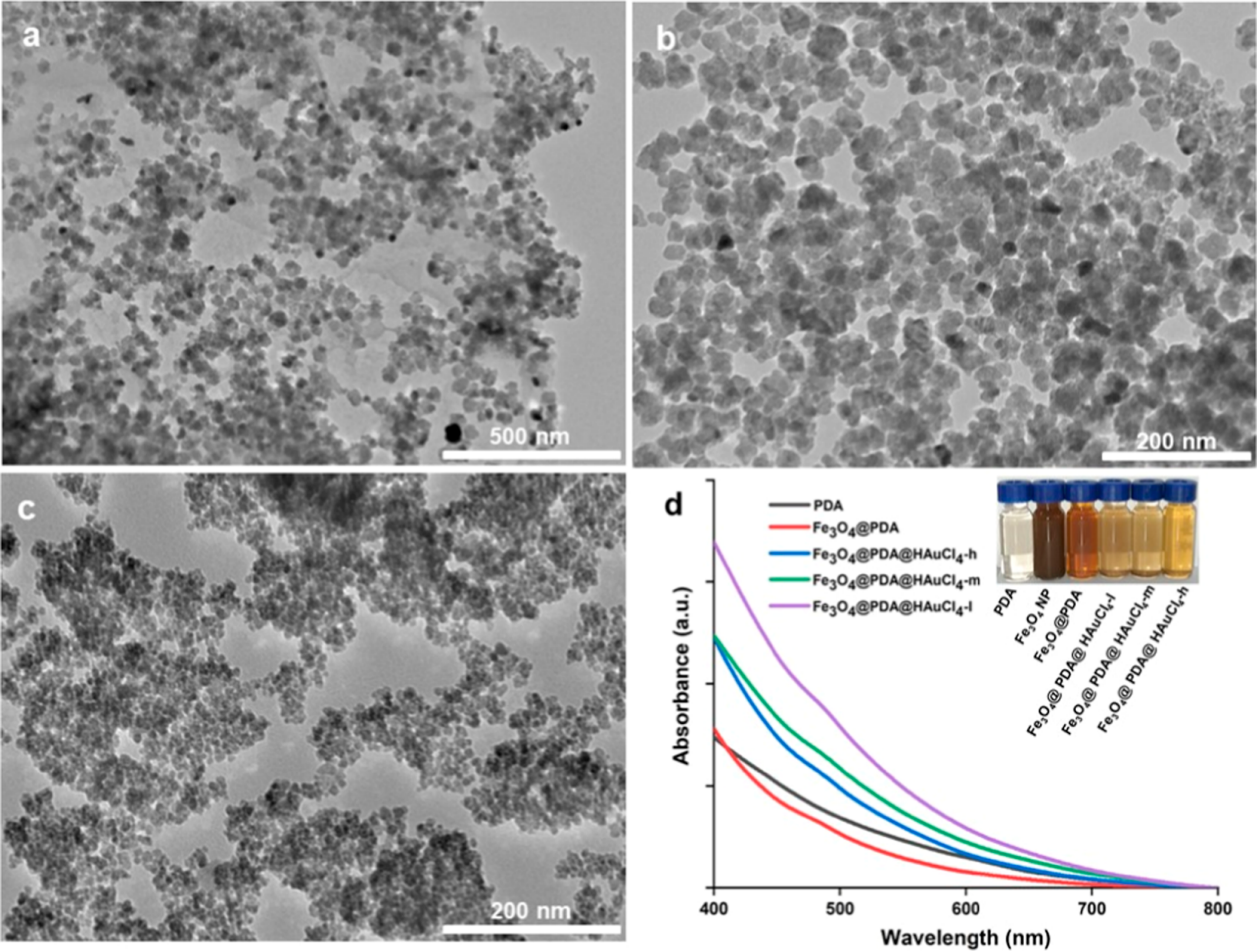
TEM images of Fe_3_O_4_@PDA@HAuCl_4_-l (a), Fe_3_O_4_@PDA@HAuCl_4_-m (b), and Fe_3_O_4_@PDA@HAuCl_4_-h (c) and UV–vis absorption spectra (d) of each nanosystem. The inset shows the optical images of NP suspensions.

As the second approach, we employed HAuCl_4_ as an Au ion source and tested the reduction of metallic ions and binding of resultant AuNPs onto the Fe_3_O_4_@PDA nanosystems. Similar to the as-prepared citrate-stabilized AuNPs’ case, the number density of deposited metal NPs was manipulated simply by tuning the amount of supplied Au ions. TEM images and absorption spectra for each resultant suspension are shown in Figure 3. Interestingly, as the amount of supplied Au ions increased, the number of AuNPs deposited onto PDA dramatically decreased (Figure 3a–c). The highest AuNP deposition was observed for the case of Fe_3_O_4_@PDA@HAuCl_4_-l which has the lowest supply of HAuCl_4_. This issue was mainly attributed to the highly acidic nature of HAuCl_4_ and the resultant low suspension pH. As depicted in our earlier studies,^30,31,40^ the low pH of colloid significantly hampers the reducing capacity of PDA which resulted in a lower number density of deposited AuNPs. This observation was further confirmed by optical images and absorption spectra (Figure 3d) of the NP suspensions. For all cases, a broad and weak shoulder was detected at around 500 nm. However, as the HAuCl_4_ supply was decreased, the intensity value was significantly increased, depicting the high number of AuNPs. For this approach, the quantity of deposited AuNPs exhibited a strong correlation with its Au ion supply and could be readily adjusted by manipulating the experimental settings. To quantify the Au content, we employed EDX spectra (Figure S4) for some representative NP systems and the results are summarized in Table 1. The increase in the supply of AuNPs (from Fe_3_O_4_@PDA@AuNP-l to Fe_3_O_4_@PDA@AuNP-h) led to an increase in the Au/Fe weight ratio from 1.48 to 1.59. For the case of Fe_3_O_4_@PDA@HAuCl_4_ NP systems, the higher Au content was detected for lower Au ion supply (Fe_3_O_4_@PDA@HAuCl_4_-l) further confirming the TEM images and UV–vis absorption spectra in Figure 3.

**Table 1:** Zeta Potentials, Rate Constants, Au/Fe Ratios, and BET Analysis Results of Different NP Systems

NP system	zeta potential (mV)	Au/Fe^a^ (in weight)	surface area (m^2^/g)	BET analysis surface area (m^2^/g) pore volume (cm^3^/g)	average pore diameter (nm)	rate constant (k, min-1)
PDA	–42.2		169.71	0.228	5.38	0.024
Fe_3_O_4_@PDA	–33.6		154.73	0.248	6.41	0.014
Fe_3_O_4_@PDA@AuNP-h	–37.7	1.59	145.43	0.222	6.13	0.31
Fe_3_O_4_@PDA@AuNP-m	–37.1	ND	ND	ND	ND	0.29
Fe_3_O_4_@PDA@AuNP-l	–30.3	1.48	ND	ND	ND	0.14
Fe_3_O_4_@PDA@HAuCl_4_-h	–12.8	1.75	ND	ND	ND	0.33
Fe_3_O_4_@PDA@HAuCl_4_-m	–21.8	ND	ND	ND	ND	0.38
Fe_3_O_4_@PDA@HAuCl_4_-l	–20.9	1.93	83.15	0.137	6.61	0.83

a Calculated according to EDX spectra provided in Figure S4. ND: not determined.

To evaluate the colloidal stability of NPs, we collected various zeta-potential measurements, and the average values are provided in Table 1. PDA NPs exhibited significantly high potential, indicating their high stability in aqueous medium. The addition of Fe_3_O_4_ NPs and AuNPs to the PDA nanosystem significantly reduced the zeta-potential values. This decrease is mainly ascribed to the attachment of these NPs to negatively charged surface groups of PDA such as catechol. However, all Fe_3_O_4_@PDA and Fe_3_O_4_@PDA@Au nanosystems still preserved their colloidal stability for months. It must be noted that the stability of the NP suspension is the main concern that determines their usage in practical catalytic applications.

To evaluate the surface nature of NP systems, we performed BET analyses, and some major characteristics including surface area, pore volume, and average pore diameter are summarized in Table 1. PDA NPs showed a surface area of 169.71 m^2^/g, a pore volume of 0.228 cm^3^/g, and an average pore diameter of 5.38 nm, indicating their mesoporous structure and large surface area, which may provide unique advantages in catalytic applications. The employment of the Fe3O4 and Au nanostructures decreased the surface area and pore volume. However, the obtained values are still highly acceptable and show a satisfying porous structure of the NP system.^55,56^

**Figure 4:**
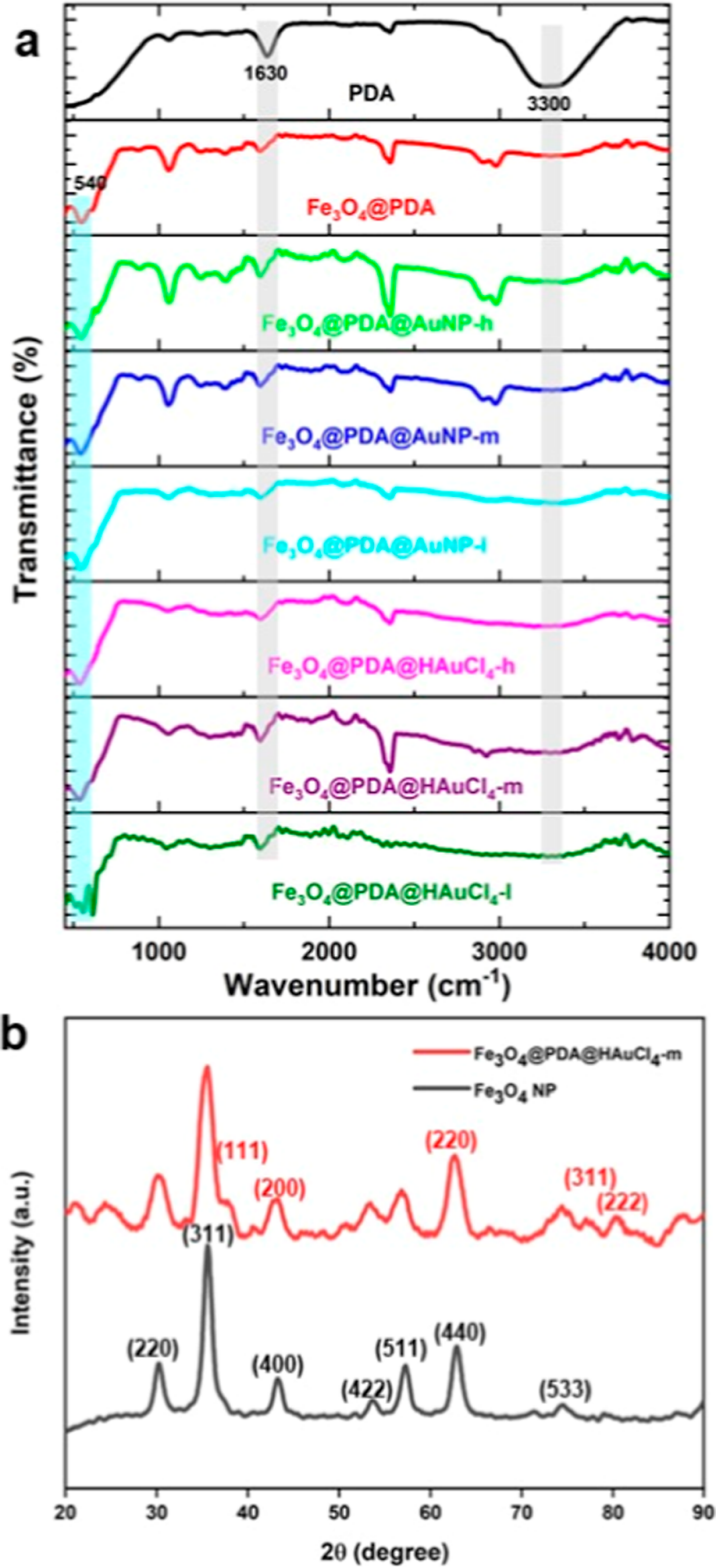
FT-IR (a) and XRD (b) spectra of NP systems.

To evaluate the chemical nature of NP systems, we collected FT-IR and XRD spectra and examined them in detail. Some informative results are shown in Figure 4. From the FT-IR spectrum of PDA NPs (Figure 4a), we observed a broad peak at around 3300 cm-1 indicating the stretching vibration associated with the N–H and broad O–H peaks.^57^ Also, remarkable bands were detected at 1630 cm^–1^ indicating the vibrational bands of the stretching and bending vibrations of N–H groups, respectively.^58^ Also, the addition of Fe_3_O_4_ NPs into the proposed system was further confirmed by the emergence of the band at 540 cm^–1^ which was related to the vibration of Fe–O.^59^ FT-IR spectra with similar bands were collected after the AuNP deposition onto the Fe_3_O_4_@PDA NP systems. The XRD pattern of Fe_3_O_4_ NPs indicated the presence of seven diffraction peaks (Figure 4b) at 30.2°, 35.6°, 43.2°, 53.7°, 57.3°, 62.8°, and 74.5° which corresponded to (220), (311), (400), (422), (511), (440), and (533) indices, respectively.^60,61^ This data is consistent with the Fe_3_O_4_ nanoparticle diffraction pattern specified in the JCPDS standard data 11–0614. The diffraction pattern revealed no diffraction peaks in any phases other than those of Fe_3_O_4_ NPs. After the deposition of AuNPs, we noticed the emergence of some diffraction peaks at 38.2°, 77.4°, and 81.4° for (111), (311), and (222) indices, respectively.^62^ Also, the broadening was observed for the peaks at 44.3° and 64.9° which were assigned to (200) and (220) indices, respectively, due to overlapping with Fe_3_O_4_ NP’s peaks. These peaks show cubic phases of gold (JCPDS No. 03-0921) with the crystalline nature of AuNPs. By using the Scherrer equation to these spectra, the crystallite size of Fe_3_O_4_ and Au nanostructures in the Fe_3_O_4_@PDA@HAuCl_4_ NP system was found to be 10.8 and 9.6 nm, respectively. These results are well correlated with the TEM images shown earlier.

### 3.2 Catalytic Activity Tests

**Figure 5:**
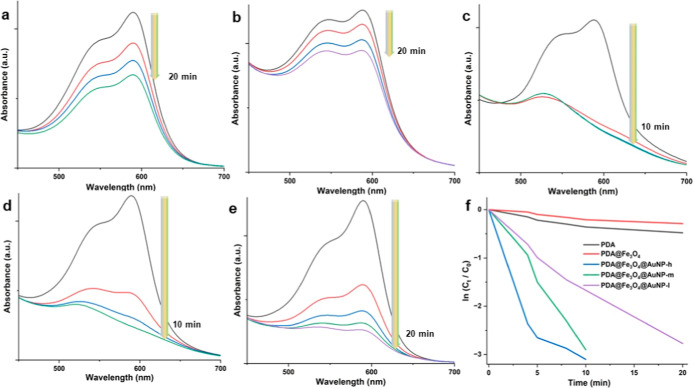
Catalytic activity tests of different NP systems for CV dye removal. UV–vis absorption spectra in the presence of PDA (a), Fe_3_O_4_@PDA (b), Fe_3_O_4_@PDA@AuNP-h (c), Fe_3_O_4_@PDA@AuNP-m (d), and Fe_3_O_4_@PDA@AuNP-l (e) and the kinetic degradation rate of CV (f).

After the detailed characterization of Fe_3_O_4_@PDA@Au nanosystems via various techniques, we tested their catalytic activity in the removal of CV and MG as dye molecules. For this, first, we employed Fe_3_O_4_@PDA@AuNP systems for the removal of CV (Figure 5). For comparison, we utilized PDA and Fe_3_O_4_@PDA nanosystems to evaluate their potential as catalysts. Interestingly, a significant decrease in the absorbance of CV was observed within 20 min in the presence of PDA and Fe_3_O_4_@PDA nanosystems (Figure 5a,b). This issue may be attributed to the opposite charge of the CV and PDA shell which led to a strong electrostatic interaction and resultant adsorption of CV onto the PDA (see Table 1).^63−65^ Also, as depicted in our earlier studies,^32,44^ a thin layer of PDA may contribute to the reduction of the dye molecule to some extent. However, the deposition of AuNPs onto the Fe_3_O_4_@PDA nanosystems created a dramatic decrease in the peak of CV at around 590 nm indicating the removal of the dye (Figure 5c–f). To quantify the kinetic data of CV degradation, we conducted several analyses using a pseudo-first-order model following the formula provided below
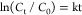
1

Here, while k is the rate constant for the pseudo-first-order model, *C*_0_ and *C*_t_ represent the initial and time-dependent concentrations of the dye molecule, respectively. The highest catalytic activity was detected for the case of the Fe_3_O_4_@PDA@AuNP-h nanosystem with a k value of 0.31 min^–1^ (we obtained this data by performing the linear fitting to the curves depicted in Figure 5f) by considering pseudo-first-order reaction kinetics (see Table 1). Fe_3_O_4_@PDA@AuNP-m and Fe_3_O_4_@PDA@AuNP-l nanosystems provided similar activities in almost complete dye removal within 10 and 20 min, respectively. It seems that as the number density of AuNPs onto the Fe_3_O_4_@PDA nanosystems was increased, the catalytic performance of the resultant system was further improved accordingly. Herein, it must be noted that the catalytic reduction of dye molecules occurs on the surface of AuNP. Therefore, the higher the number of metallic NPs, the higher the catalytic removal of dye molecules. The catalytic degradation mechanism of CV can be explained as follows: the BH^4–^ ions were hydrolyzed in the aqueous solution.^6,30−32,43,44,53^ The generated active hydrogen and electrons could reduce CV. The transport of electrons from electron donor NaBH_4_ to electron acceptor CV can be expedited by the presence of AuNPs in Fe_3_O_4_@PDA@AuNP systems. Upon reduction of CV, the reduced molecules were detached from the surface of nanosystems, initiating the subsequent catalytic reduction process. Two pathways for CV including the demethylation of amine sites of the chromophore system and the breaking of the whole conjugated chromophore skeleton can be proposed for catalytic degradation.^66^ Lastly, the presence of Fe_3_O_4_@PDA@AuNP was noticed from absorption spectra after the completion of the reaction without any significant change, confirming the robustness and stability of NP systems.

**Figure 6:**
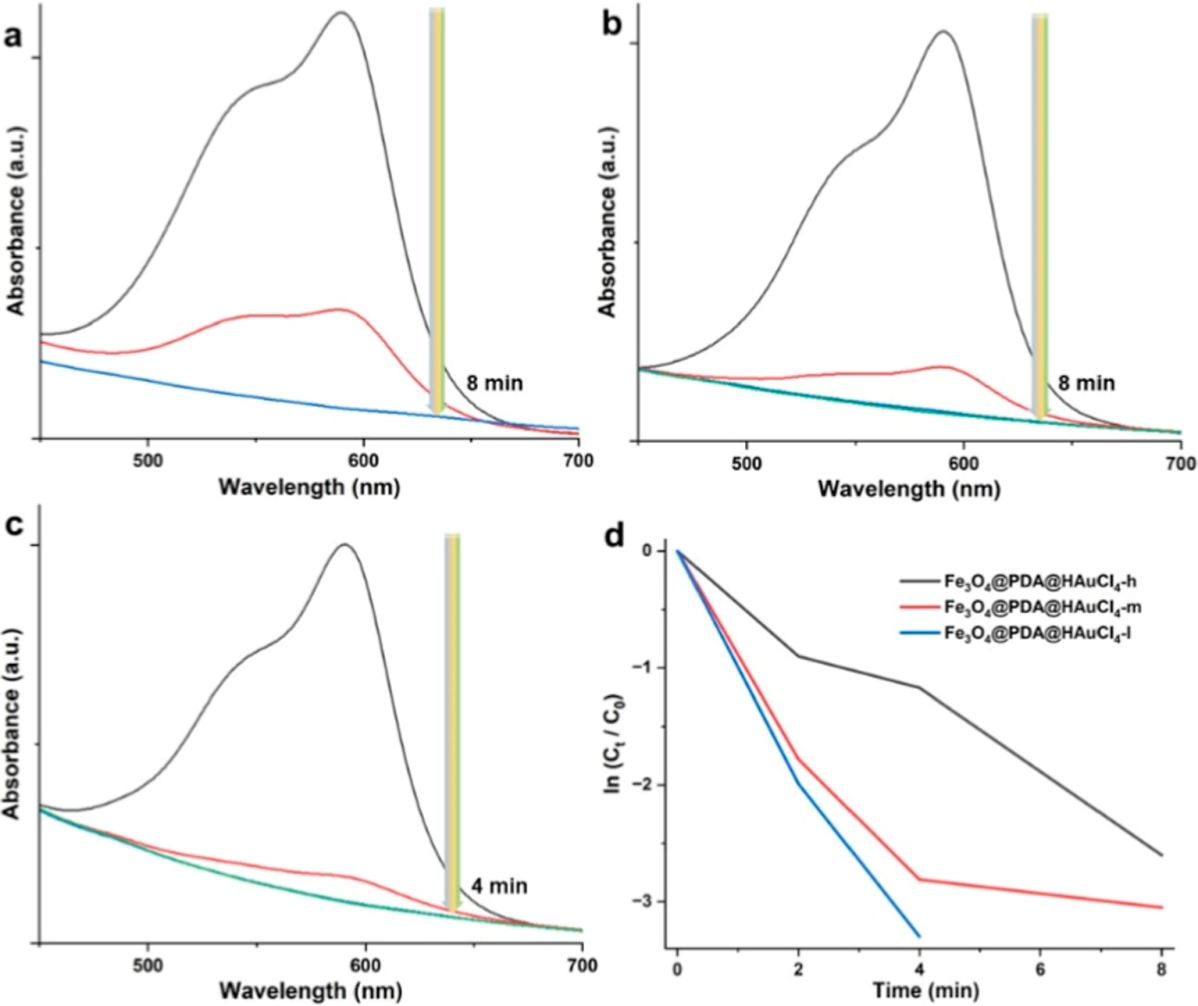
Catalytic activity tests of different NP systems for CV dye removal. UV–vis absorption spectra in the presence of Fe_3_O_4_@PDA@HAuCl_4_-h (a), Fe_3_O_4_@PDA@HAuCl_4_-m (b), and Fe_3_O_4_@PDA@HAuCl_4_-l (c) and the kinetic degradation rate of CV (d).

For the catalytic tests, we utilized a similar strategy to test the catalytic activity of the Fe_3_O_4_@PDA@HAuCl_4_ nanosystems (Figure 6). For the case of Fe_3_O_4_@PDA@HAuCl_4_-l, complete dye removal of CV was attained only within 4 min (Figure 6c). However, both Fe_3_O_4_@PDA@HAuCl_4_-h and Fe_3_O_4_@PDA@HAuCl_4_-m nanosystems could perform the complete dye degradation within 8 min. The rate constants for Fe_3_O_4_@PDA@HAuCl_4_-h, Fe_3_O_4_@PDA@HAuCl_4_-m, and Fe_3_O_4_@PDA@HAuCl_4_-l were calculated from the slope of the curves shown in Figure 6d and found to be 0.33, 0.38, and 0.83 min^–1^ (see Table 1), respectively. The relatively higher catalytic activity of Fe_3_O_4_@PDA@HAuCl_4_ NP systems may be attributed to the smaller size of deposited AuNPs with a high number density.^67^ The high number of NPs with smaller sizes could create more active sites for catalytic conversion and an improved reduction capacity for dye removal.

**Figure 7:**
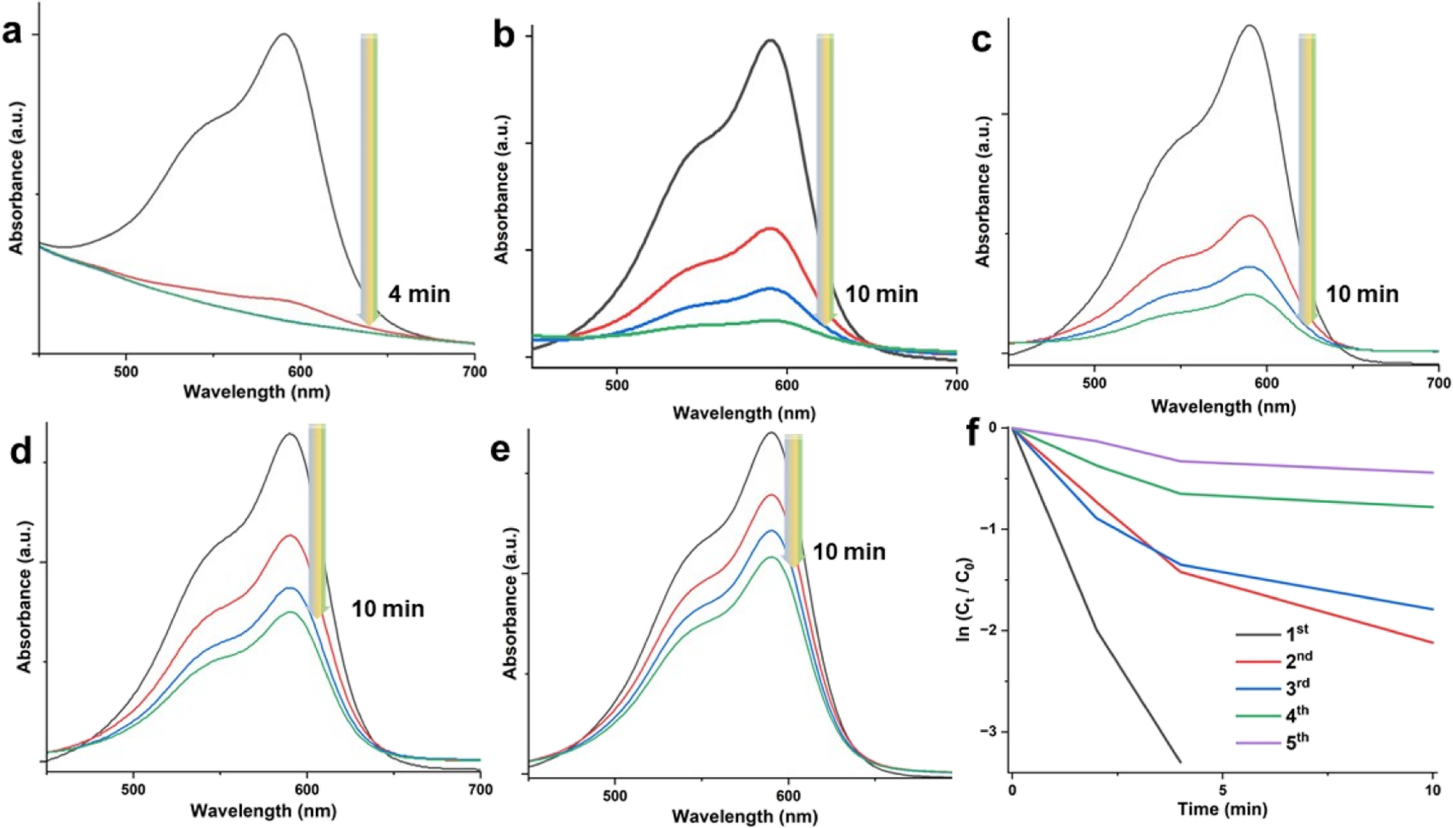
Reusability test of the Fe_3_O_4_@PDA@HAuCl_4_-l NP system for CV dye removal. 1st (a), 2nd (b), 3rd (c), 4th (d), and 5th (e) recycle and the kinetic degradation rate of CV (f).

**Figure 8:**
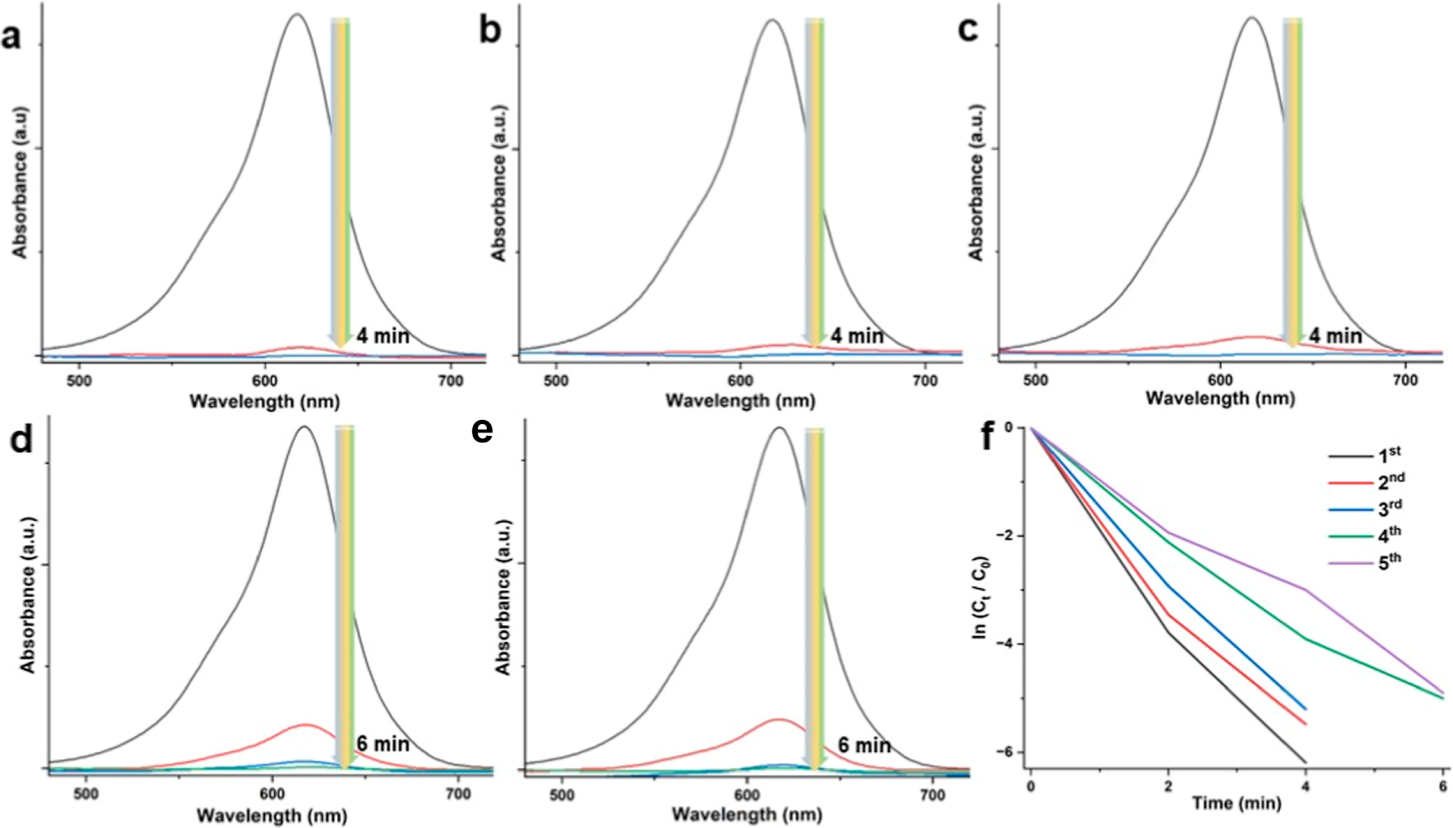
Reusability test of the Fe_3_O_4_@PDA@HAuCl_4_-l NP system for MG dye removal. 1st (a), 2nd (b), 3rd (c), 4th (d), and 5th (e) recycle and the kinetic degradation rate of MG (f).

In most cases, the long regeneration time and low cyclic stability are major limitations of PDA-based dye removal systems.^5,68^ These issues create many challenges in their employment as an ideal reusable adsorbent as well as a catalyst. Therefore, we performed various studies to test the regeneration and reusability capacities of the proposed nanosystem. For this, we selected the Fe_3_O_4_@PDA@HAuCl_4_-l NP system and employed it in consecutive catalytic applications. First, after the first cycle of dye removal, NPs could be completely removed from the solution in less than 7 min (see the video in the Supporting Information). The removed NPs were washed with DI water for 3 min and they were ready to use for the next test. To show the universality of the NP system, their regeneration and reusability ability were tested in the removal of CV and MG dyes. The results of these analyses are shown in Figures 7 and 8. For the case of CV, even after the fifth cycle (Figure 7e), 36% of dye molecules were catalytically eliminated within only 10 min. The further increase in the reaction time for this cycle indicated the continuous degradation in a similar trend, but it was not shown here for clarification. In 120 min, the complete dye removal was attained for this case which is still a satisfying performance compared with similar studies in the literature (see and compare Table 2). The decrease in the catalytic activity of the NP system may be due to many issues including the probable mass losses during the regeneration procedure, the reduction in the adsorption capacity of PDA, and the desorption of AuNPs from the surface.^64^ In the case of MG, we could obtain a unique performance in terms of regeneration and recyclability (Figure 8). For the first three recycles, the NP system provided 100% dye removal in only 4 min (Figure 8a–c) with a k value of 1.54 min^–1^ (see Table 2). The usage of the Fe_3_O_4_@PDA@HAuCl_4_-l NP system in the fourth and fifth recycles (Figure 8d,e) completes dye removal in only 6 min which is still a highly acceptable catalytic performance. The suggested degradation mechanism for MG is the assault of hydroxyl radicals on the central carbon, leading to the breaking of the C–C bond and *N*,*N*-dimethylaminobenzyl which produce *p*-dimethylaminophenol and 4-(*N*,*N*-dimethylaminomethyl)benzylone as intermediates. These intermediates are then converted into benzoic acid, *p*-(dimethylamino)-benzoic acid, and hydroquinone.^69^ In comparison to CV, the higher dye removal rate of MG can be ascribed to various parameters such as chemical structure, nature and charge of the dye molecule, and resultant adsorption efficiency onto the NP surface.^64^

**Table 2:** Catalytic Activity of Different NP Systems with Similar Content for Dye Removal

catalytic system	dye molecule(s)	rate constant(s) (min–1)	reference
Fe_3_O_4_@PDA-Ag	methylene blue (MB)	0.34	(6)
Fe_3_O_4_@PDA-Ag	MB	0.43	(5)
Fe_3_O_4_@Au/PDA	4-nitrophenol (4-NP)	0.41	(3)
PS@Fe_3_O_4_/PDA@Ag	4-NP	0.41	(67)
urchin-like Fe_3_O_4_@PDA-Ag hollow microspheres	MB	1.78	(70)
Fe_3_O_4_@PDA@AuNP-h	CV	0.31	this study
Fe_3_O_4_@PDA@HAuCl_4_-l	CV	0.83	
Fe_3_O_4_@PDA@HAuCl_4_-l	MG	1.54	

## 4 Conclusions

In conclusion, we have developed a simple yet effective method to fabricate Fe_3_O_4_@PDA@Au nanosystems in the core/shell structure. The PDA shell exhibited excellent adsorption against positively charged dye molecules and played an important role in both attachment of as-prepared AuNPs and reduction of Au ions. The change in the amount of both supplies of AuNPs or Au ions enabled the deposition of the Au nanostructures onto Fe_3_O_4_@PDA in a well-controlled manner. For both approaches, the resultant NP systems exhibited unique catalytic activities with *k* values of 0.83 and 1.54 min^–1^ for the dye removal of CV and MG, respectively. We noticed that the amount of AuNPs deposited was the main factor that governed the resultant catalytic activity of the proposed system. Also, with their unique regenerable characteristics, Fe_3_O_4_@PDA@Au nanosystems exhibited satisfying recyclability even after the fifth recycle. Our results undoubtedly suggest that the proposed core/shell nanosystem has great potential in various applications including catalysis and wastewater treatment.

## Supporting Information Available

Nanoparticle regeneration (MP4)
Representative TEM image of the PDA NP system; UV–vis absorption spectra of AuNPs; particle size distributions of AuNP, Fe_3_O_4_, and PDA NP systems; and EDX spectra of some NP systems including Fe_3_O_4_@PDA@AuNP-l, Fe_3_O_4_@PDA@AuNP-h, Fe_3_O_4_@PDA@HAuCI_4_-l, and Fe_3_O_4_@PDA@HAuCI_4_-h (PDF)
