# Supplementary material for: Regenerable Poly(dopamine)-Mediated Gold Nanostructure-Decorated Core–Shell Nanostructures of Magnetite/Polydopamine for Catalytic Dye Removal
Source: ACS Omega. 2024 Dec 18;9(52):51163–74. doi: 10.1021/acsomega.4c06968 (PMC11696424; doi:10.1021/acsomega.4c06968)
Supplement: Supplementary file 2 — ao4c06968_si_002.pdf [file ao4c06968_si_002.pdf]

Supporting Information

Regenerable poly(dopamine)-mediated gold nanostructure-decorated core‒shell nanostructures of magnetite/polydopamine for catalytic dye removal

Nuray Serginay[1+], Mehmet Semih Bingol[2+], Erkan Karatas[3], Mehmet Yilmaz[1,2,4*]

[1]Department of Nanoscience and Nanoengineering, Atatürk University, Erzurum, Turkiye

[2]East Anatolia High Technology Application and Research Center (DAYTAM), Atatürk University, Erzurum, Turkiye

[3]Department of Molecular Biology and Genetics, Erzurum Technical University, Erzurum, Turkiye

[4]Department of Chemical Engineering, Atatürk University, Erzurum, Turkiye

*Corresponding author

mehmetylmz@atauni.edu.tr

+ equally contributed authors

Content

Figure S1. Representative TEM image of PDA NP system.

Figure S2. UV-vis absorption spectra of AuNPs.

Figure S3. Particle size distributions of AuNP (a), Fe_3O_4 (b), and PDA (c) NP systems.

Figure S4. EDX spectra of some NP systems. Fe_3O_4@PDA@AuNP-l (a), Fe_3O_4@PDA@AuNP-h (b), Fe_3O_4@PDA@HAuCI$_4$-l (c), and Fe_3O_4@PDA@HAuCI$_4$-h (d).

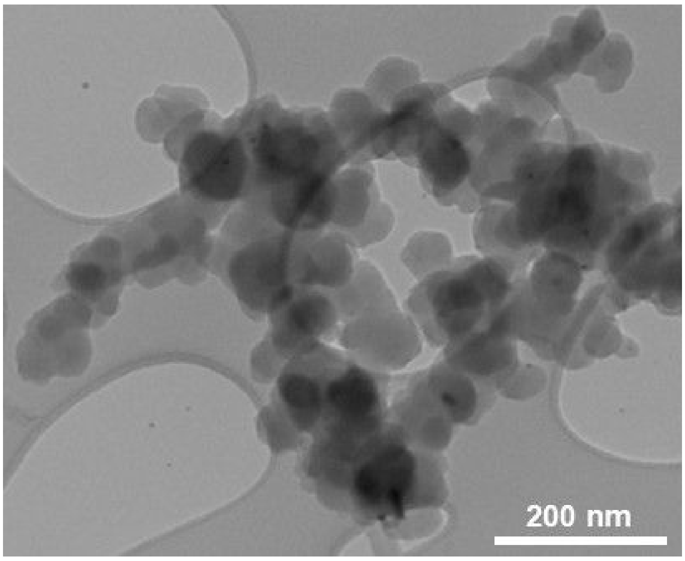

Figure S1. Representative TEM image of PDA NP system.

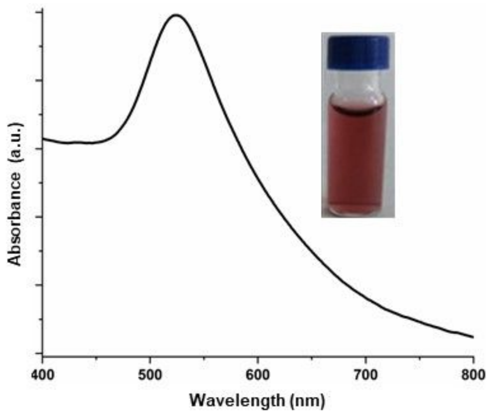

Figure S2. UV-vis absorption spectra of AuNPs. The inset shows the optic image of AuNP suspension.

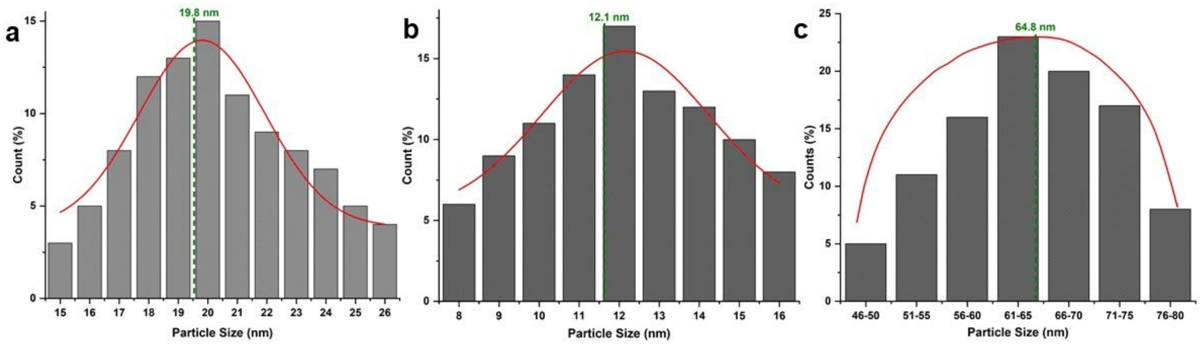

Figure S3. Particle size distributions of AuNP (a), Fe_3O_4 (b), and PDA (c) NP systems.

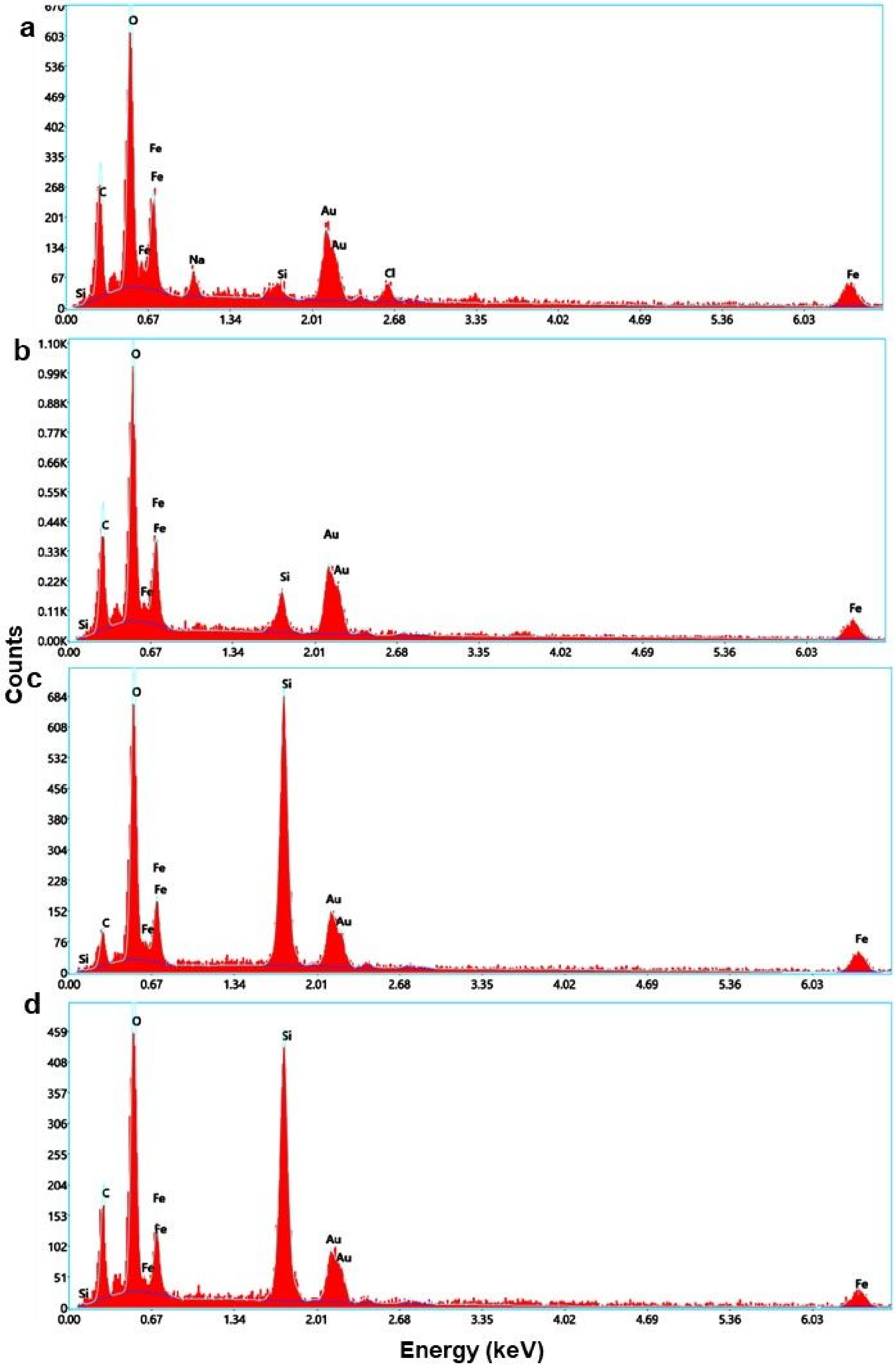

Figure S4. EDX spectra of some NP systems. Fe_3O_4@PDA@AuNP-l (a), Fe_3O_4@PDA@AuNP-h (b), Fe_3O_4@PDA@$HAuCl_4$-l (c), and Fe_3O_4@PDA@$HAuCl_4$-h (d).
